# Of the Effect of Magnesia in Promoting the Solubility of Peruvian Bark; with Some Account of What Is Called the Essential Salt of Bark

**Published:** 1786

**Authors:** Thomas Skeete


IV. *Of the Effect of Magnesia in promoting the Solubility of Peruvian Bark; with some Account of what is called the Essential Salt of Bark.*—Vide *Experiments and Observations on Quilled and Red Peruvian Bark* †. *By* Thomas Skeete, *M. D.* 8vo. London, 1786.

IT was the late Dr. Macbride who first observed ‡ that lime, triturated with resinous gums, promotes their dissolution in water; but as lime communicates a very disagreeable taste to tinctures prepared in this manner, Mr. Henry was induced, many years ago, to try how far this solubility might be promoted by employing calcined magnesia instead of lime. He was led to this by considering the great affinity which

† For the remainder of the title see the Catalogue of Books.

‡ Experimental Essays, 2d edit. p. 238.

this ſubſtance is known to have with fixed air, and the circumſtance of its being itſelf inſoluble in water, and of courſe incapable of giving any particular impregnation to the tinctures in which it might be employed. He accordingly tried it with camphor, opium, gum guaicum, and other reſins *. The following is the account he gives of its effect on the bark:

"A drachm of Peruvian bark, twenty grains "of calcined magneſia, and four ounces of "diſtilled water, being rubbed together du-"ring fifteen minutes, the filtered infuſion re-"ſembled, in appearance, the ſimple tincture "of bark, but was not ſtrongly impregnated "with the peculiar aroma of the bark †."

Dr. Skeete, conſidering this action of magneſia on Peruvian bark as a curious and intereſting circumſtance which had not been ſufficiently attended to, was induced to proſecute the ſubject, and, for this purpoſe, made the following experiments:

* Bergman alſo, in ſpeaking of calcined magneſia, has mentioned its effect in promoting the ſolubility of reſinous ſubſtances in water.—See his *Opuſc. Phyſ. et Chemic.* Vol. I.

† Experiments and Obſ. (chap. viii.) on the ſolvent qualities of calcined Magneſia. 8vo. London, 1773.

Experiment I.—Two drachms of Peruvian bark in powder, and half a drachm of calcined magnesia, which had lost more than half its weight by calcination, were rubbed together in a mortar, with four ounces of distilled water, for the space of ten or fifteen minutes; the water being gradually added, so as to reduce the materials, in the first instance, to the state of a paste. The infusion, when passed through filtering paper, was found to be possessed of the following remarkable properties:

1st. It had an exceedingly deep red colour, superior to that of the infusion of common bark in lime water.

2d. It was more bitter and astringent to the taste even than an infusion of red bark.

3d. It produced a very deep black colour, with a copious precipitation, upon the addition of a solution of *sal martis*; while a similar addition to a common infusion of bark occasioned a moderate discoloration and small precipitation only.

4th. It remained beautifully transparent three or four days, and was so strongly antiseptic, that at the end of a week, in summer, it had scarcely made any advances towards fermentation; tion; while an infuſion of bark, with ſimple water, fermented in two days.

5th. It exceeded in ſpecific gravity the infuſion of bark in lime water, in the ſame, or rather in a greater proportion, than the latter exceeds the ſimple infuſion.

In order to determine more particularly the nature of the infuſion prepared by this experiment, ſeveral additions were made to different portions of it. Being mixed in equal quantities with water impregnated with fixed air, no other effect was produced than that of ſimple dilution. A ſmall quantity of the acid of ſugar, however, being added to ſome of the infuſion, immediately diſcharged the red colour, and cauſed a whitiſh precipitation: hence our author thinks it is obvious, that magneſia not only increaſes the activity of water upon bark, but that it is in fact diſſolved itſelf in the water in a very ſmall proportion.

Calcined magneſia, added to an infuſion of bark, prepared in the common way with ſimple water, occaſioned no change in its colour or properties. From this circumſtance, our author thinks we may conclude, that when bark and magneſia are rubbed together with water, in the manner before mentioned, the magneſia

 either either enables the water to extract something from the bark, which it could not have done alone, or, what is more probable, that, by uniting chemically, they form a compound more active and soluble in water than pure bark.

With a view of ascertaining how far the colouring matter of an infusion of bark with magnesia corresponds with the astringency of it, our author made the following experiment:

EXPERIMENT II.—The clear and colourless liquor was carefully poured off from the precipitate, which the acid of sugar had occasioned when added to the infusion of bark and magnesia, and being mixed with a proper quantity of the chalybeate solution, changed to a green colour only; from which circumstance, Dr. Skeete thinks it probable there is a close connexion between the colouring matter and astringency; for he has found that the deeper the red colour of the infusion, the more complete always is the black which the chalybeate produces.

Magnesia, he observes, differs remarkably from lime in its action upon bark; for, whether it be added in a small or large quantity, it promotes the solution, though it does this more completely in proportion as the quantity is greater. By the addition of half a drachm, or a drachm at the utmoſt, however, to two drachms of bark and four ounces of water, the full effects, he tells us, are obtained, ſo that an additional quantity of the magneſia would only be waſted.

It was next an object with our author to try the action of common magneſia upon bark, in order to determine how far the preſence or abſence of fixed air could aſſiſt in the explanation of the effects which have been enumerated.

Experiment III.—One drachm of common magneſia was rubbed in a mortar fifteen minutes with two drachms of bark, and four ounces of pure water, in a ſimilar manner to the infuſion with calcined magneſia, and being filtered, was ſubjected to all the trials which were made with that infuſion. Some little difference was perceived in favour of the infuſion with calcined magneſia; but the other, we are told, exhibited ſimilar properties in every reſpect.

Dr. Skeete acknowledges, that if two infuſions be prepared, the one with half a drachm of calcined, the other with the ſame quantity of common magneſia, the former will appear much ſtronger, the proportion of real magneſia being double; but when allowance is made for the preſence of fixed air in common magneſia, all the effects, he is perſuaded, may be obtained from it nearly, if not in an equal degree, with the calcined.

From this experiment Dr. Skeete thinks we are authorized to conclude, that fixed air has no ſhare in the changes produced by the action of magneſia and bark on each other; but at the ſame time we find him candidly acknowledging that he is unable to account for them in a ſatiſfactory manner. He, therefore, contents himſelf with having aſcertained facts, which he thinks may be applicable to uſeful purpoſes, and he leaves the explanation of them to others, or to ſome future opportunity, when he may have leiſure to reſume his inquiries on this ſubject.

It is worthy of obſervation, that, in our author's experiments, neither common nor calcined magneſia, when added to bark and water, and agitated in a vial, or even when boiled together, produced the effects which have been mentioned; the particles of the magneſia being ſo light, that they cannot be made to act upon thoſe of the bark, unleſs well rubbed in a mortar mortar to the confiftence of a pafte, previous to the addition of the whole quantity of the water.

Several experiments were inftituted by our ingenious author, with the view of obferving the effects of heat on bark and magnefia; but in none of the trials did the action of the latter on the bark appear to be increafed by heat.

With regard to the medicinal properties of an infufion of bark with magnefia, Dr. Skeete informs us, that the accounts he has received from his medical friends, who have adminiftered it, have been as fatisfactory as the nature of the fubject will permit.—Its rich red colour, the tranfparency it preferves for three or four days, and the length of time it remains found, without the addition of any fpirituous water, are qualities, he thinks, which fhould operate in obtaining it an extenfive application to medical purpofes. He himfelf confiders it as an excellent fubftitute for the decoction and infufion of the red bark.

But befides employing magnefia in infufion with bark, our author propofes that they fhould be given together more frequently in fubftance than has hitherto been the practice. Some phy-ficians, he obferves, have occafionally prefcribed fcribed powdered bark and magnefia, with a few grains of the aromatic fpecies, but with no other expectation from the magnefia than that of obviating coftivenefs; from his inquiries on this fubject, however, Dr. Skeete feems to think that the efficacy of the bark would be increafed by fuch a mode of exhibition.

We fhall conclude this article with the account Dr. Skeete gives in another part of his work, of the *effential falt of bark*, as it is called, prepared and fold by Mr. Godfrey, of Southampton Street, and which our author fuppofes to be a very delicate extract, procured entirely free from empyreuma by means of the water bath. It is made to affume a curious plated appearance, fomewhat refembling fhell-lac, though much thinner. — The complete and ready folution of this preparation in the faliva, and in water, while it is but moderately affected by proof fpirit, and not at all by rectified, would feem, our author thinks, to indicate the prefence of a quantity of the mucilage of gum arabic; but whether this be the cafe or not, there is every reafon, he obferves, to believe that it is poffeffed of confiderable efficacy.

